# Long term changes in aquaculture influence migration, regional abundance, and distribution of an avian species

**DOI:** 10.1371/journal.pone.0284265

**Published:** 2023-04-13

**Authors:** Paul C. Burr, Brian S. Dorr, Jimmy L. Avery, Garrett M. Street, Bronson K. Strickland

**Affiliations:** 1 Mississippi Field Station, National Wildlife Research Center, Wildlife Services, Animal and Plant Health Inspection Service, United States Department of Agriculture, Mississippi State, Mississippi, United States of America; 2 National Warmwater Aquaculture Center, Mississippi State University, Stoneville, Mississippi, United States of America; 3 Department of Wildlife, Fisheries, and Aquaculture, Mississippi State University, Mississippi State, Mississippi, United States of America; MARE – Marine and Environmental Sciences Centre, PORTUGAL

## Abstract

Agricultural development has been causing changes to the environment and the abundance and distribution of avian species. Agriculture is dynamic with changes in products occurring at large scales over relatively short time periods. The catfish aquaculture industry is one such agriculture industry that has undergone dramatic changes over the last 25 years. The double-crested cormorant (*Nannopterum auritum*) is a piscivorous bird that has an extensive history with the aquaculture industry of Mississippi due to its depredation of cultured catfish. A large-scale monitoring program began in 1989 to estimate the abundance and location of cormorants at every known roost in the primary catfish producing region of the state, regionally known as the Delta. We used this data set to address hypotheses pertaining to cormorant ecology within the Delta over time, particularly in relation to aquaculture. We found that, although the Midwest breeding population of cormorants has been increasing, the abundance of cormorants wintering in the Delta has been decreasing, closely following the decline of aquaculture, suggesting aquaculture area is the primary reason for cormorant inhabitation of the region. We also modeled cormorant presence and abundance at all roost sites to determine what factors most influenced cormorant distribution. Aquaculture area around roosts was a significant predictor of both cormorant presence and abundance. However, the influence of aquaculture area was seasonally dependent, with greater positive influences occurring prior to migration. Lastly, we found peak cormorant abundance in the Delta is occurring 2.14 days earlier each year, which may be indicative of changes to migration phenology. Information gained using this large dataset aids in cormorant damage mitigation and to further our understanding of cormorant ecology. Data indicate changes in agriculture, and potentially climate change, can influence phenology, distribution, and abundance of avian species at large geographic scales.

## Introduction

The development of agriculture has been a driving force in causing changes to the environment [1, 2]. By 2000, 55% of Earth’s ice-free land had been converted into cropland, pasture, and urban areas [2]. Agriculture production is dynamic, with crops produced and their production practices changing over time to cause local and regional increases in some crops and declines in others. Market forces, changes in production practices [3], and climatic change [4] influence these changes in agriculture. Agriculture has transformed landscapes and is one of the greatest causes of change to wildlife populations worldwide [1, 3]. In most cases, this change in habitat has been negative for wildlife, but in some instances wildlife benefit from agriculture-dominated landscapes [5].

Considerable research has been conducted on impacts of agriculture on avian species [1, 3, 5]. Less often reported are the beneficial aspects of agriculture on avian populations, although some have been documented. For example, one study found granivorous bird species populations increased in the long term with intensive row crop agriculture [5]. Less common are studies examining impacts of declines in largescale agriculture beneficial to avian species, on those avian species. An agriculture product that has gone through relatively recent and rapid change is commercial production of catfish (*Ictalurus* spp.). Growth of the catfish industry in the U.S. has been closely tied to increases in abundance of fish-eating birds, particularly the Double-crested cormorant (*Nannopterum auritum*; hereafter, cormorant) [6, 7].

**Fig 1:**
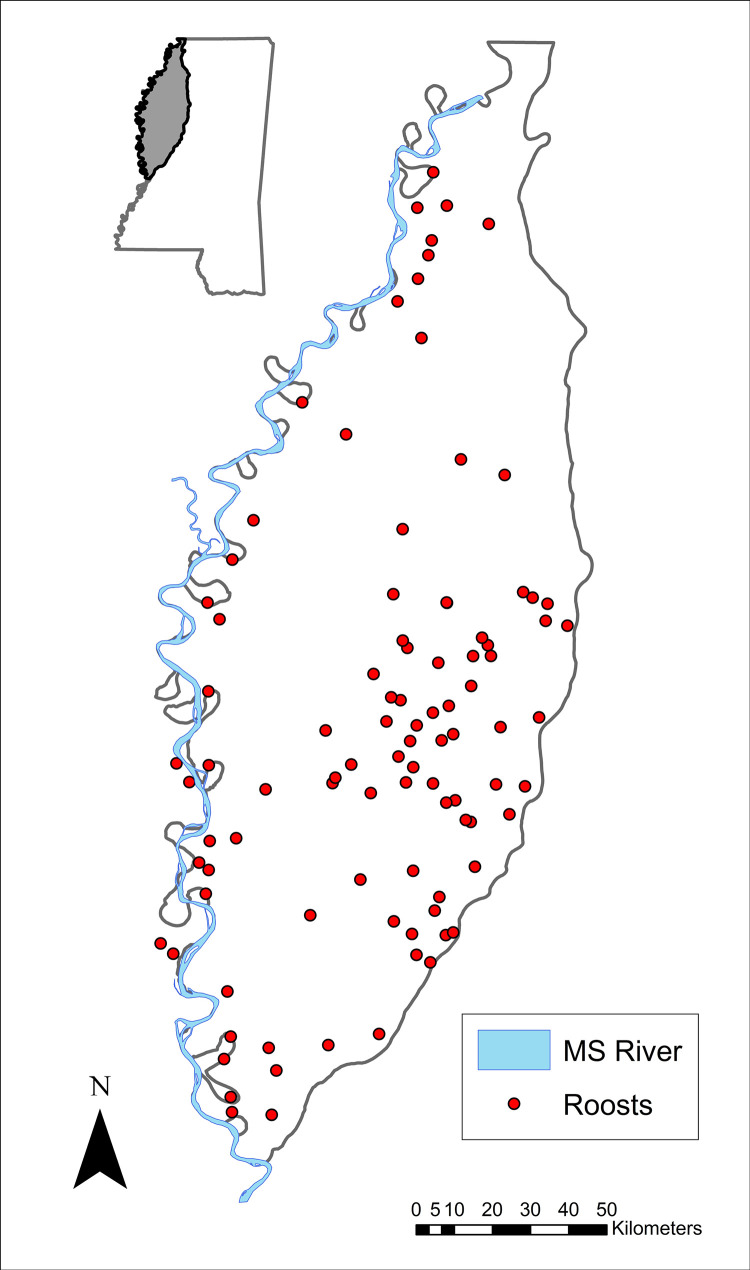
Portion of the Mississippi alluvial valley located in Mississippi, known as the Mississippi Delta. Abundance data of cormorants at roosting sites (red points) were collected by USDA WS-NWRC during winter months (October–April) beginning in 1989. Mississippi state outline, Mississippi Delta outline, and the Mississippi river shapefiles were downloaded from Mississippi Automated Resource Information System (MARIS) at Maris.mississippi.edu. Mississippi river shapefile credit: U.S. Geological Survey and National Geospatial Program with no use limitations. Mississippi state and Mississippi Delta shapefile credit: U.S. Census and MARIS with no use limitations. Roost location shapefile was created using spatial information data from roost surveys, collected by the U.S. Department of Agriculture, Wildlife Services. This figure was created in ArcGIS.

Commercial production of catfish is the largest aquaculture industry in the U.S., and most production (59%) occurs in Mississippi [8]. Most of Mississippi’s catfish aquaculture is located within an 18,000 km^2^ region located in the northwest portion of the state, known as the Mississippi Delta (Fig 1; hereafter Delta) [9]. The Delta lies near the terminus of the Mississippi Flyway, a major migratory route for many avian species in North America, including cormorants. Cormorants are a colonial waterbird widely distributed across North America [10], typically breeding in the northern U.S. and southern Canada during warmer months, and wintering in the southern U.S. and Mexico [11]. Cormorants diet primarily consists of fish but can also include crustaceans, amphibians, reptiles, and small mammals [10]. Although cormorants are currently well established throughout their range and listed as a species of ‘least concern’ [12], their historic population has fluctuated greatly.

Cormorants have a long history of conflict with humans through the depredation of fish resources [13, 14]. Persecution of cormorants first began during European settlement in the 19^th^ century to reduce competition over fish species [14, 15]. Cormorant populations began to steadily decline thereafter as control efforts intensified, habitat alterations were made, and pesticides were introduced into the environment [10, 16]. Populations were reduced so severely the species was listed as ‘special concern’ in several states in the U.S. during the 1970s [17]. However, the cormorant population began to recover shortly thereafter [15, 18–20]. This recovery has been attributed to the inclusion of cormorants in the Migratory Bird Treaty Act, the ban of DDT use in 1972, and the growth of aquaculture industries throughout the U.S. [15, 21].

Although the recovery of cormorants has been successful, public perception of the species remains controversial [22]. Active management of cormorants continues due to their possible impact on commercial and natural resources [23]. For example, cormorants can have adverse effects on vegetation [24, 25], displace other avian species [26–28], and potentially compete for fishery resources used for recreational purposes [23, 29]. However, of particular concern are the impacts cormorants have on the catfish aquaculture industry [30].

Historically, most of the cormorant population wintered along the Atlantic and Gulf of Mexica and were infrequently found on fresh waters of Mississippi [31]. The abundance of cormorants wintering inland in the southeastern U.S. steadily increased during the latter part of the 20^th^ century [15, 20]. Evidence suggests this increased abundance coincided with the increase in catfish aquaculture in the Delta [6, 7] and was likely driven by benefits derived from this rich foraging resource. For example, cormorant use of catfish aquaculture has been shown to increase fitness correlates like pre-migratory omental fat reserves [32]. The increased abundance and use of catfish aquaculture caused concern among catfish producers over the economic loss associated with bird depredation at their facilities [30]. Consequently, a large-scale monitoring program was implemented by U.S. Department of Agriculture, Wildlife Services, National Wildlife Research Center (WS-NWRC) beginning in 1989 to estimate the abundance of cormorants at all roost locations throughout the Delta (Fig 1).

Counts of cormorant numbers at roosts have given ecologists and wildlife managers relative abundance estimates used to study the biology and behavior of cormorants, typically as it relates to the aquaculture industry [33–35]. Numerous studies exist on methods to disperse cormorants from roosts and their effectiveness to reduce depredation of cultured catfish [36–40]. Some basic information has also been gleaned from these studies with respect to cormorant roost ecology. For example, cormorants spend approximately sixty percent of their time at roost sites, and only eighteen percent foraging in Mississippi [41], making suitable roost locations a necessity in the species’ daily activity and home range. Roosts are typically permanently flooded forest wetland consisting of bald cypress (*Taxodium distichum*) and occasionally tupelo gum (*Nyssa aquatica*) [42]. Although there appears to be short-term roost fidelity, it is also reported that cormorant roost usage fluctuates throughout the winter season [42, 43]. Winter home ranges for cormorants are relatively large, averaging approximately 17,490 km^2^ [44], and cormorants can travel upwards of 33 km from their night roost to subsequent foraging sites [41]. The highly mobile nature of cormorants allows them to select from many available roosting sites found throughout the Delta during the winter months, but a comprehensive understanding of the mechanism driving cormorant distribution and abundance in Mississippi is lacking. Using the long-term data set collected by the WS-NWRC provided us the opportunity to explore cormorant ecology and behavior over a large spatial and temporal extent. Specifically, we addressed factors influencing cormorant distribution at roosting sites in the Delta, annual trends of cormorant abundance in the Delta, and phenology of maximum cormorant abundance in the Delta.

## Materials and methods

### Data description

WS-NWRC has collected two types of cormorant roost abundance data over the years. The first was mid-winter roost counts, in which every known active roost was surveyed by an individual, or individuals, who directly counted cormorants from the ground or water by boat. Approximately half of all roosts were surveyed for 3 hours before sunset, and the other half were surveyed the following morning for 3 hours after sunrise. Mid-winter roost counts were only done once per year, typically in early February, when cormorant abundance in the Delta was assumed to be at its maximum [34]. This large-scale effort was logistically challenging and involved numerous volunteers to count each roost. However, the abundance estimates derived from counts were an accurate estimate to the actual number of cormorants present within the entire Delta at the time of the survey as all roosts were monitored within a 24-hour period. Twenty-five years of mid-winter roost counts were available, including 1989–2010, 2012, and 2016–2017.

The second type of roost abundance data were aerial surveys, in which a pilot flew a fixed wing aircraft at an altitude of 100–150 m over each known roost location, and an observer recorded all cormorants present at the roost. Approximately half of the roosts were surveyed within 4 hours after sunrise and the other half surveyed within 4 hours before sunset [43]. Counts were made during these times because cormorants tend to remain at roosts during the early morning and late evening hours [10, 42]. All aerial surveys were done within a single 24-hour period to avoid double counting, and multiple surveys were conducted over the winter months of each year, typically twice per month from October through April, coinciding with cormorant migration movement through Mississippi [20, 37]. Compared to the mid-winter roost count, aerial surveys serve more as a relative index of cormorant abundance as each roost was only briefly surveyed. Eighteen years of aerial surveys were available, including 1996–2010, and 2015–2017. During the last three years of collecting aerial survey data (2015–2017), individual observers corrected for error associated with their counting by taking digital photographs of cormorants at a subset of roosts. Observers systematically photographed roosts to ensure a range of counts were covered [37], and photographs were manually counted and modeled against aerial counts using linear regression, while keeping the intercept set to zero [6]. We built separate regression models for each of the three winters and multiplied each model’s slope with its respective winters count. We used the averaged slope from these models as an overall correction factor, which equaled 1.29, by multiplying it with all other aerial survey counts. The value of our correction factor was similar to other avian surveys that took place in the Delta. For example, a correction factor of 1.25 was used to correct observer bias when surveying wintering ducks from the air [45].

We used these large data sets provided by WS-NWRC to address the following specific objectives pertaining to cormorant population ecology in the Delta. Information gained from addressing these objectives will improve our knowledge of cormorant ecology in the state of Mississippi, particularly in relation to aquaculture, and inform future management strategies therein. First, we examined cormorant distribution at roosting sites in the Delta. Our goal was to determine spatiotemporal distribution patterns of cormorants wintering in the Delta in relation to roost characteristics and surrounding aquaculture. Second, we examined annual trends in cormorant abundance in the Delta. We compared annual observed cormorant abundance in relation to changes in aquaculture area as well as in relation to the Midwest breeding cormorant population. Third, we explored possible variation in the phenology of maximum cormorant abundance in the Delta by quantifying if the timing of maximum abundance has changed annually.

### Statistical methods

#### Cormorant distribution at roosting sites in the Mississippi Delta

In 2017, the Delta contained 89 known cormorant roosts, but the number of active roosts has generally increased since the surveys began. For example, in 1996 approximately 55 roosts were known and surveyed. Roosts were added or removed either when new roosts were discovered, or existing roosts were drained or converted. We have a general understanding of how the cormorant population was distributed throughout the Delta for given time periods over the winter season of many years using precise roost locations and cormorant counts from aerial surveys. We wanted to examine how cormorant distribution throughout the Delta behaves on a seasonal and annual scale, and in relation to roost surroundings, including changes in aquaculture production (S1 Dataset).

We used a two-step modeling approach to separately model cormorant presence/absence at roosting sites, and positive abundances [46]. This approach helps handle the typical count data scenario in which excess zeros are present by modeling the occurrence and abundance processes separately [47]. First, the presence model was used to model the binary response of cormorant presence at a roost (1 = present, 0 = absent).Second, a zero truncated model was used to model positive count values [48, 49]. The separation of presence and abundance models allows us to investigate the processes governing each independently and allows the incorporation of different variables hypothesized to influence each.

We modeled cormorant presence at roosting sites by fitting a generalized linear mixed model (GLMM) with a binomial distribution using the *lme4* package [50] in R version 3.6.2. Similarly, we modeled cormorant abundances at roosting sites by fitting a GLMM with a negative binomial distribution. We included roost ID as a random effect in both models to account for unmeasured variables associated with each roost and because multiple data points were taken [47]. We first identified three fixed effects related to forage potential around each roost to include in both the presence and abundance models. Fixed effects included the distance from the roost to the Mississippi river, and the area of aquaculture and area of natural water bodies within a 23.4 km buffer around each roost. The 23.4 km buffer is the median distance cormorants move to subsequent day locations [40], and we therefore treated the area within this buffer as the space available to cormorants occupying roosts. For each roost during each year, we calculated the area (ha) of both catfish aquaculture and naturally occurring water bodies within their buffers by manually digitizing all aquaculture area and natural water bodies using multispectral satellite imagery in a geographic information system (ArcGIS v10.2). This multispectral satellite imagery (30-m resolution) was taken from one of the Landsat satellites (5, 7, or 8), curtesy of the U.S. Geological Survey (USGS), and obtained from USGS Earth Explorer. We used a multi-band method combining spectral bands 5, 6, and 4 (Landsat 8) or 4, 5, 3 (Landsat 5 and 7), and displayed them as red, green, and blue, respectively, to improve surface water detection [51].

We predicted both aquaculture area and natural water body area would have a positive influence on cormorant presence and abundance, as greater forage availability was likely favorable. Cormorants roosting near the Mississippi river have less catfish in their diet, presumably due to the differences in foraging habitat availability [52]. We therefore included distance to the Mississippi river to measure possible preference toward roost sites in relation to the river. However, these three foraging variables were highly correlated. Distance to the Mississippi river and natural water body area showed a negative relationship (r = -0.86, t = -203.07, df = 14,974, *p* < 0.001), as naturally occurring water body area was concentrated near the river. Likewise, catfish aquaculture was concentrated in the east-central region of the Delta, farther away from the river (r = 0.43, t = 57.53, df = 14,974, *p* < 0.001), and therefore with less natural water body area (r = -0.55, t = -81.29, df = 14,974, *p* < 0.001). Our primary interest was understanding cormorant distribution and abundance in relation to aquaculture as their interaction continually cause human-wildlife conflict in the Delta. We therefore chose to keep the variable of aquaculture area in the models and neither natural water body area nor distance to the Mississippi river.

Cormorant abundance in the Delta fluctuates throughout the winter season as migration activities occur [34]. We included nominal date (where October 01 = 01, October 02 = 02, etc.) as a third order polynomial in both the presence and abundance models to account for this seasonal variation. We also included an interaction term between nominal date and aquaculture area in both models to examine seasonal variations of cormorant distribution and abundance in relation to foraging potential, hypothesizing foraging behavior would be dynamic over the winter season. Because the act of flying is one of the greatest energy expenditures for cormorants [53], we predicted cormorants would show higher probability of use and greater abundances at roosts with greater amounts of aquaculture within their buffers later in the winter season, before northerly migration occurs.

We also included the number of other known cormorant roosts found within the 23.4 km buffer of each roost for the presence model. Other cormorant roosts may offer cormorants an alternative location in the event a disturbance or other perceived risk was present at their currently selected roost and predicted roosts with more alternative roosts within their buffer would have higher probability of use. We included year and the area of the roost itself in the abundance model. The area of a roosting site (ha) has been thought to influence cormorant use, but results vary [42, 43]. We predicted larger roosts would have a positive influence on cormorant abundance as they typically have more trees available to perch. The available roost survey data indicates annual cormorant abundance in the Delta has generally been decreasing, so we included year as a quadratic term in the model to account for changing regional population size which will influence the average number of cormorants at any given occupied roost.

We standardized all continuous variables prior to modeling both presence and abundance data to aid in model convergence and parameter estimation [54]. We standardized variables by subtracting each data value by the mean of all values, and then dividing by its standard deviation. The resulting standardized variables all have a mean of 0 and standard deviation of 1. We also checked for spatial autocorrelation of the count data by calculating Moran’s I for each of the 209 roost surveys [55]. Only 9 surveys (4.3%) showed possible evidence of autocorrelation (*p* < 0.05) and we elected to use all data in the analysis. We used the package *effects* in R to calculate means and confidence intervals to graphically display independent variables’ influence on presence and abundance of cormorants at roosting sites [56]. Resulting plots were created by predicting the model’s response while allowing the variable of interest to vary over its range and holding all other variables at their mean.

#### Annual trends of cormorant abundance in the Mississippi Delta

We examined annual fluctuations of cormorant abundance in the Delta against the regions changing aquaculture industry. Cormorant population size wintering in the Delta was suggested to be correlated with the size of the states’ aquaculture industry[10, 43, 57]. As aquaculture growth began to slow in the late 1990s, the apparent growth of wintering cormorants in the Delta declined as well [43]. We hypothesized the number of cormorants wintering in the Delta to be closely related to the food availability (primarily surface area of aquaculture present) in the region. The aquaculture industry began in the 1960’s, peaked in the early 2000’s, and has steadily decreased thereafter [58, 59]. Therefore, we predicted cormorant abundance in the Delta would follow a similar pattern. If true, the Delta may currently have fewer total cormorants occupying the region than past studies have estimated (e.g., [42, 57]), as cormorants that once occupied, or may have occupied the Delta may continue their migration through the southeastern states to coastal habitat where they have wintered in the past [10, 31].

The abundance of cormorants in, or migrating through, the Delta is likely related to the overall migratory population size. Cormorants that move through the Delta throughout the winter originate from the Upper Midwest United States and Prairie Pothole region of Canada including much of the Great Lakes [10, 60]. Cormorants typically begin their fall migration in early October, following the Mississippi Flyway south [11]. Therefore, we calculated a breeding bird survey index as a general estimate of the Midwest cormorant breeding population for each year of data to be used in our analysis. This breeding bird survey index was created by totaling all breeding cormorants recorded in the bird conservation regions 8, 11, 12, 13, 22, and 23 using breeding bird survey data from 1989 to 2017 [61]. We ran a Pearson’s correlation test between the breeding bird survey index and year to determine if the breeding population shows signs of either growth or decline.

We selected the maximum recorded abundance from either the mid-winter roost count or the aerial survey data to serve as our dependent variable for each year of cormorant roost abundance data. Because the mid-winter roost count is generally a more thorough estimate (each roost is observed for multiple hours), it routinely produces a greater count than any aerial survey count within the same year. However, the mid-winter count was only done once per year and the resulting cormorant abundance was dependent on the date of the survey, cormorant migration timing, temperature, etc. Therefore, if during any year there was a greater aerial survey count observed we elected to use that count value. We also ran a Pearson’s correlation test between maximum aerial count and midwinter roost count to justify the use of either value (S2 Dataset).

We used linear regression to model the maximum recorded cormorant abundance per year against aquaculture water surface area present within the Delta and the breeding bird survey index of that year. Total aquaculture area and breeding bird survey were not significantly correlated (r = -0.22, t = -1.18, df = 24, *p* = 0.25) and were therefore both included in model construction. We modeled both response variables as either linear or quadratic terms to allow for possible non-linear relationships. We selected the best model based on Akaike information criterion, corrected for small sample size (AICc) [62].

We predicted both the breeding bird survey index and aquaculture area would be influential on the maximum number of cormorants observed in the Delta. To measure which variable was more influential on the dependent cormorant count, we used package *relaimpo* in R [63]. This package computes the relative importance of each predictor by computing their R^2^ contribution to the model, while averaging over all combinations of orderings among the regressors [63].

### Phenology of maximum cormorant abundance in the Mississippi Delta

The phenology of avian behavior has received a great deal of attention, particularly in relation to climate change [64, 65]. We explored possible changes in the timing of the maximum cormorant abundance in the Delta over time. This period reflects the most intense use of a migratory stopover at a critical pre-migratory period in preparation for northerly migration to the cormorant’s breeding grounds [32]. Maximum cormorant abundance in the Delta also poses the greatest concern for catfish producers and maximizing efforts to reduce cormorants on or near aquaculture sites during this time is important. Additionally, any annual changes in this timing may warrant further investigation, possibly in relation to climate change [66].

We used similar methodology described by previous studies to model cormorant abundance within the Delta region for each year of aerial survey data [34]. Cormorant counts were modeled against nominal date, and counts at the beginning (October 01) and end (April 30) of the winter season were assumed to be zero. We used polynomial terms of nominal date progressing from second order up to sixth order [34]. We did not consider first order models as the cormorant population in the Delta would not grow or decline linearly within a year as individuals will migrate to, and eventually from, the region. For each year we chose the lowest polynomial model in which the R^2^ value did not substantially change in the succeeding polynomial model [34] (i.e., < 0.01). We then determined the date corresponding to the maximum modeled abundance for each year. We modeled dates against year using a linear regression model to determine if the date associated with maximum cormorant abundance in the Delta has changed over time (S3 Dataset).

This research, including field methods and data collection, was approved under U.S. Department of Agriculture, Wildlife Services, National Wildlife Research Center Quality Assurance protocol, QA-2322, including Institutional Animal Care and Use and attending vet approvals.

## Results

### Cormorant distribution at roosting sites in the Mississippi Delta

**Fig 2:**
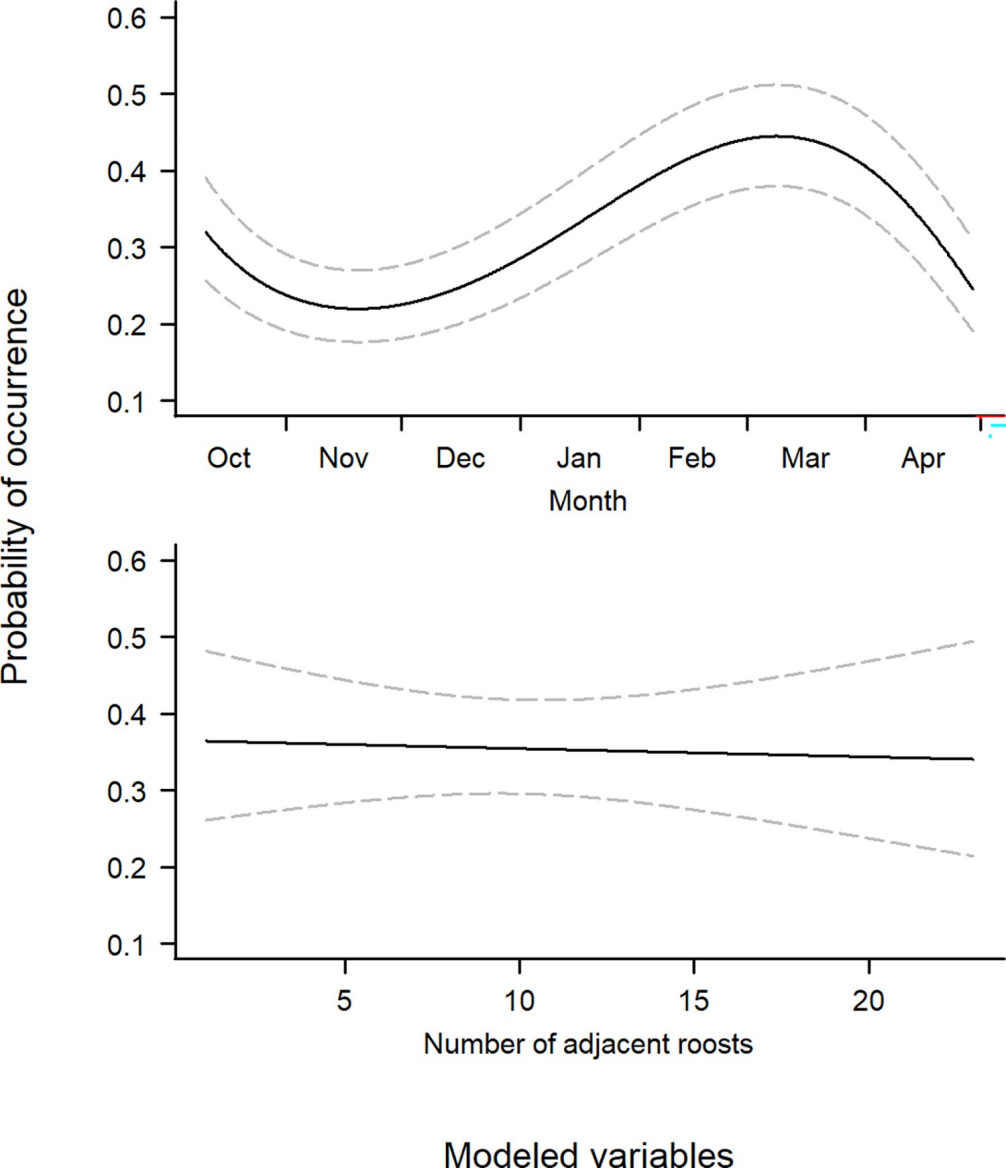
Predicted occurrence probability (+ 95% CI) of cormorants at roosting sites in the Mississippi Delta in relation to date, and the number of other roosts within a 23.4 km buffer. Trend lines were created from model predictions made by allowing each variable of interest to vary over its range and holding all other variables at their mean.

**Fig 3:**
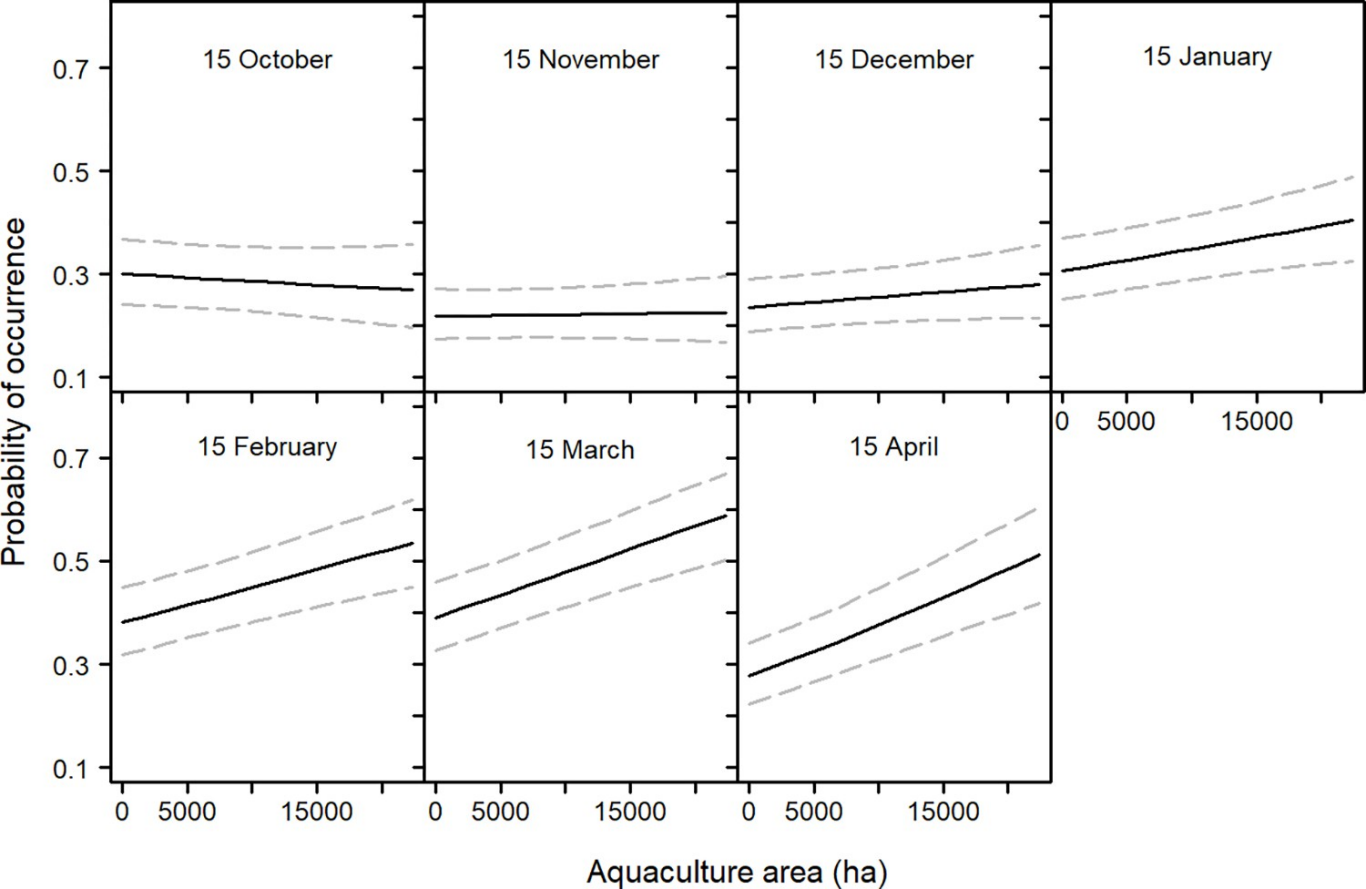
Predicted occurrence probability (+ 95% CI) of cormorants at roosting sites in the Mississippi Delta in relation to an interaction term of aquaculture area within a 23.4 km buffer and date. Trend lines were created from model predictions made by allowing each variable of interest to vary over its range and holding all other variables at their mean.

All 18 years of aerial survey data were included in the analysis, with an average of 11.6 (SD: 3.8) surveys conducted per year. The presence model showed aquaculture area within the 23.4 km buffer to have a positive influence on cormorant presence at the central roosting site. The nominal date variable revealed cormorant presence at roosts peaked in February through March (Fig 2). The interaction between aquaculture area and nominal date showed aquaculture area to have less of an influence on use probability in the earlier months of winter, but gradually increases through April (Fig 3). Lastly, the number of other roosts within the 23.4 km buffer did not show a significant influence on the probability of cormorant roost use (Fig 2 and Table 1).

**Table 1:** Parameter estimates for standardized variables modeled for cormorant presence and positive count values at roosting locations in the Mississippi Delta. The presence model was done using generalized linear mixed effects model with a binomial distribution based on cormorant presenece and absence. The count model was done using a generalized linear mixed effects model with negative binomial regression on counts greater than zero. For both models roost ID was set as a random effect. Data was collected at multiple time points over the winter months (October–April) from 1996–2010, and 2015–2017.

Model Variable^a^	β	SE	p-val
Presence Model^b^			
Intercept	-0.68	0.14	
Date	35.90	2.29	< 0.001
Date^2^	-6.39	2.35	0.007
Date^3^	-27.27	2.30	< 0.001
Other Roosts	-0.03	0.14	0.836
Aquaculture area	0.15	0.04	0.001
Aquaculture area: date	0.11	0.02	< 0.001
Abundance Model^c^			
Intercept	6.12	0.10	
Year	-30.12	1.96	< 0.001
Year^2^	12.56	1.64	< 0.001
Date	-16.72	1.51	< 0.001
Date^2^	-30.00	1.50	< 0.001
Date^3^	-9.71	1.47	< 0.001
Roost area	0.46	0.12	0.001
Aquaculture area	0.09	0.06	0.096
Aquaculture area: date	0.21	0.02	< 0.001

^a^Variable Descriptions

Date: Nominal date where October 1^st^ = 01 for each year of data; Other Roosts: Number of other roosts within a 23.4 km buffer; Aquaculture area: area of aquaculture, measured in hectares, within a 23.4 km buffer; Year: year of data collection, including 1996–2010 and 2015–2017; Roost area: area of the roost, measured in hecatres.

^b^ Delta AIC of null presence model = 424.8

^c^ Delta AIC of null abundance model = 783.6

**Fig 4:**
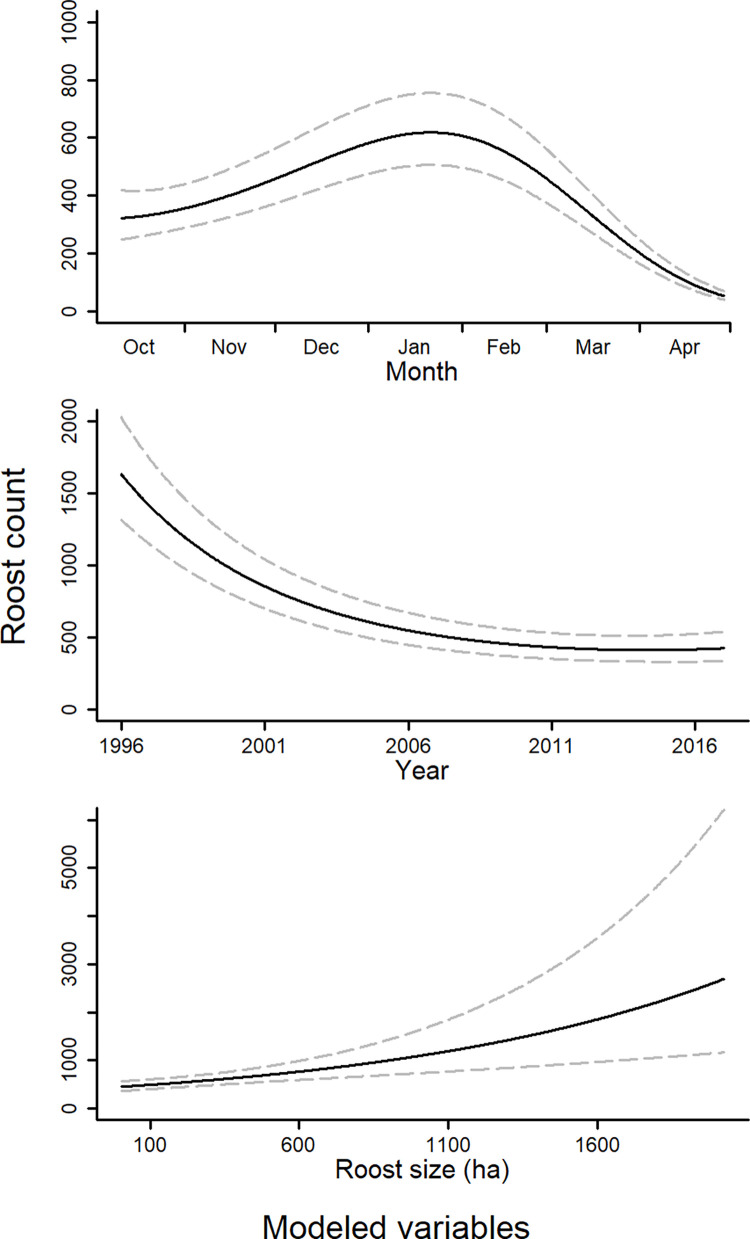
Average predicted abundance (+ 95% CI) of cormorants at roosting sites in the Mississippi Delta in relation to date, year, and the area of the occupied roost. Trend lines were created from model predictions made by allowing each variable of interest to vary over its range and holding all other variables at their mean.

**Fig 5:**
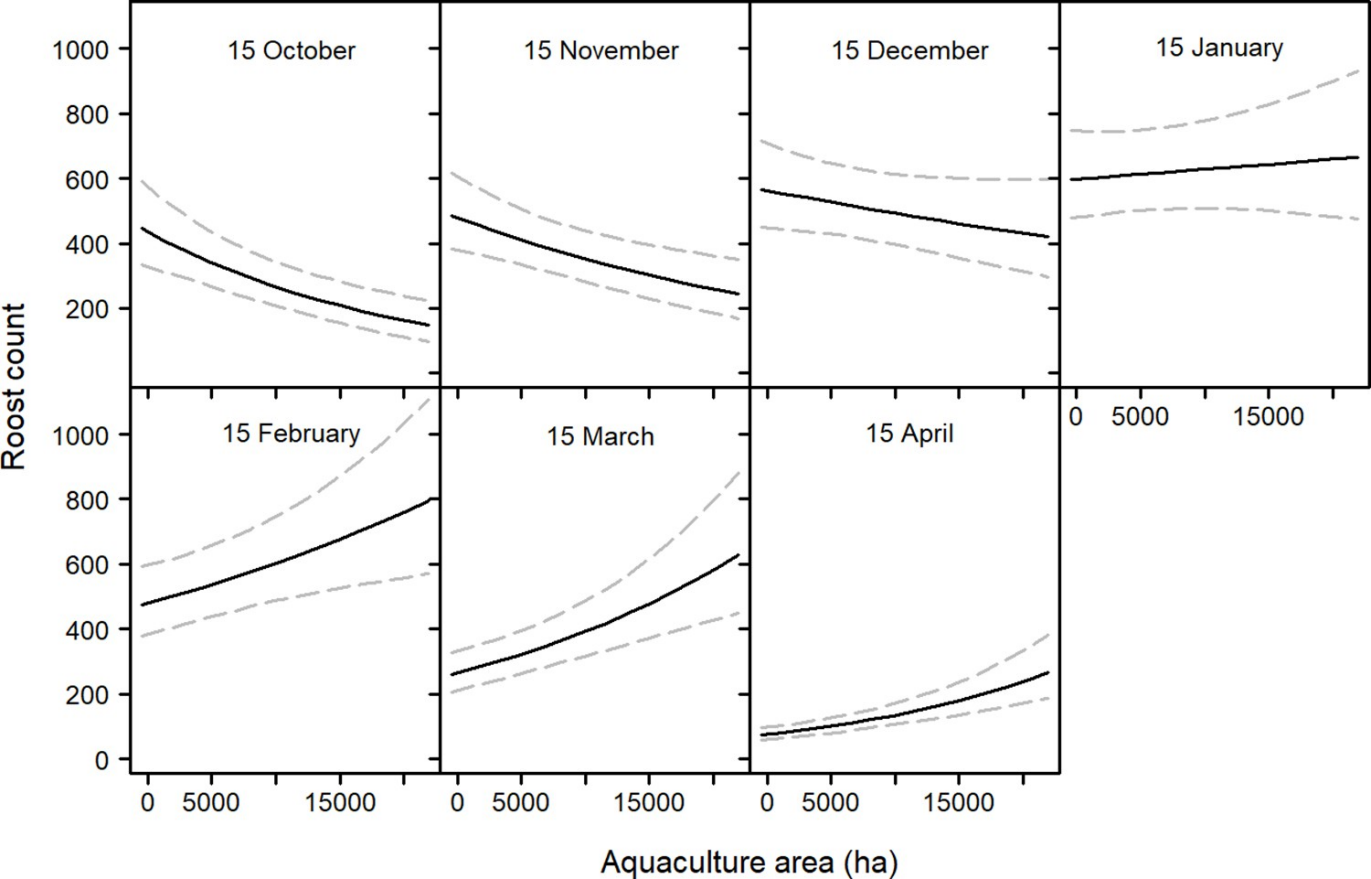
Average predicted abundance (+ 95% CI) of cormorants at roosting sites in the Mississippi Delta in relation to an interaction term of aquaculture area within a 23.4 km buffer and date. Trend lines were created from model predictions made by allowing each variable of interest to vary over its range and holding all other variables at their mean.

The count model showed average roost abundance increases from October through January, and steadily declines thereafter (Fig 4). The main effect of aquaculture area was not significant, but the interaction of aquaculture area and nominal date was (Table 1). Aquaculture area has a negative influence on cormorant abundance at a roost in the early months of winter, whereas the opposite was observed toward the end of winter (Fig 5). Average roost abundance shows a general decrease since the beginning of our data set (1996), but appears relatively constant in more recent years. Lastly, roost area showed a positive influence on average abundance (Fig 4).

#### Annual trends of cormorant abundance in the Mississippi Delta

**Fig 6:**
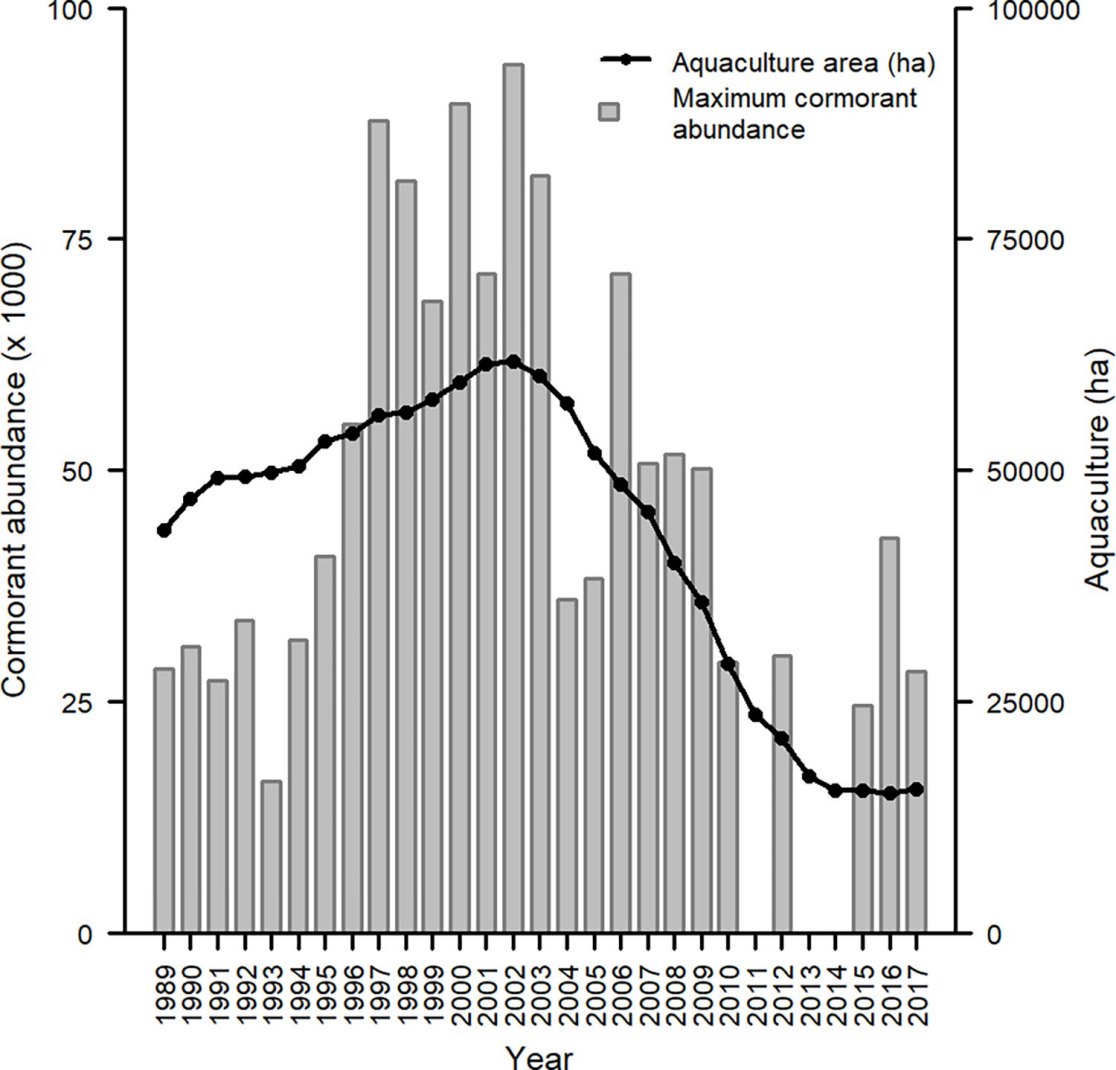
Maximum recorded cormorant abundance and total aquaculture area within the Mississippi Delta from 1989 to 2017.

Maximum aerial survey count and midwinter roost count were positively correlated (r = 0.76, t = 4.54, df = 15, *p* < 0.001). Out of the 16 years of available aerial survey count data, five years had higher maximum counts compared to midwinter roost counts (1997, 1998, 2000, 2002, 2006), and in 2015 no mid-winter count was done so the maximum aerial count was used. Twenty-six years of data were included in the analysis, including 1989–2010, 2012, and 2015–2017 (Fig 6). Our breeding bird survey index ranged from 195 to 1198 and was positively correlated with year (r = 0.55, t = 3.40, df = 27, *p* = 0.002), indicating the breeding population of cormorants within the selected bird conservation regions has been increasing since 1989. The greatest observed cormorant abundance in the Delta occurred from 1997 to 2003 (mean count per year = 79,315), which coincided with the greatest amount of aquaculture area ever present in the Delta (Fig 6).

**Fig 7:**
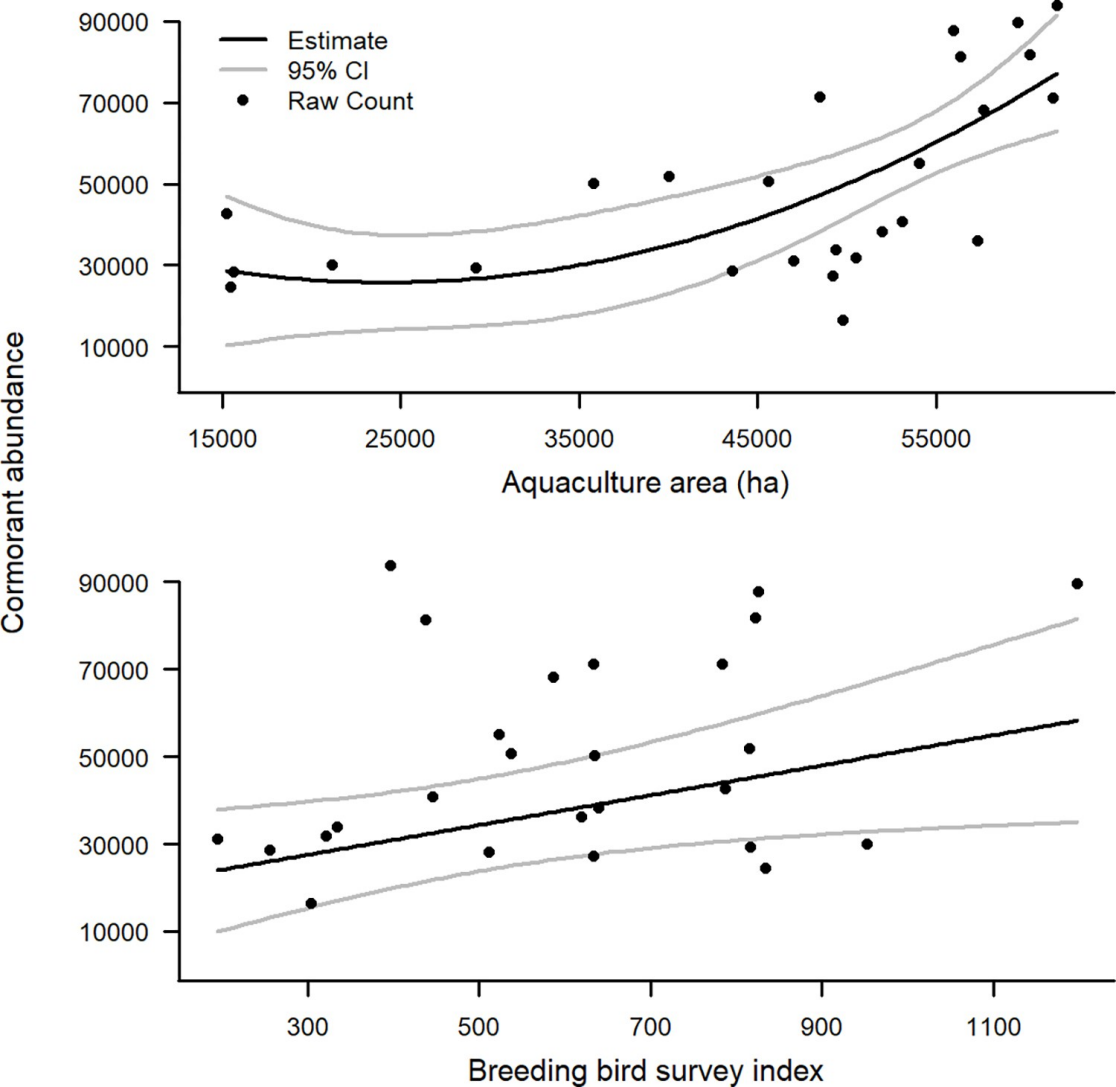
Modeled relationship (+ 95% CI) of total aquaculture area in the Mississippi Delta and breeding bird survey index on the maximum observed cormorant abundance within the Mississippi Delta. Estimates were made using linear regression by modeling yearly maximum cormorant abundance against both predictor variables, from 1989–2010, 2012, and 2015–2017. Top ranked model included aquaculture area as a quadratic term and breeding bird survey index as a linear term.

The top model ranked by AICc included breeding bird survey index as a linear term and aquaculture area as a quadratic term for predicting maximum observed cormorant abundance. The R^2^ for this model was 0.61, with the breeding bird survey index accounting for 18.4% of the total R^2^, and aquaculture area accounting for 81.6%. Each variable had a positive influence on maximum observed cormorant count. Lower values of aquaculture area showed less influence on cormorant abundance compared to larger values, whereas breeding bird survey displayed a gradual positive influence on maximum observed cormorant abundance (Fig 7).

### Phenology of maximum cormorant abundance in the Mississippi Delta

**Fig 8:**
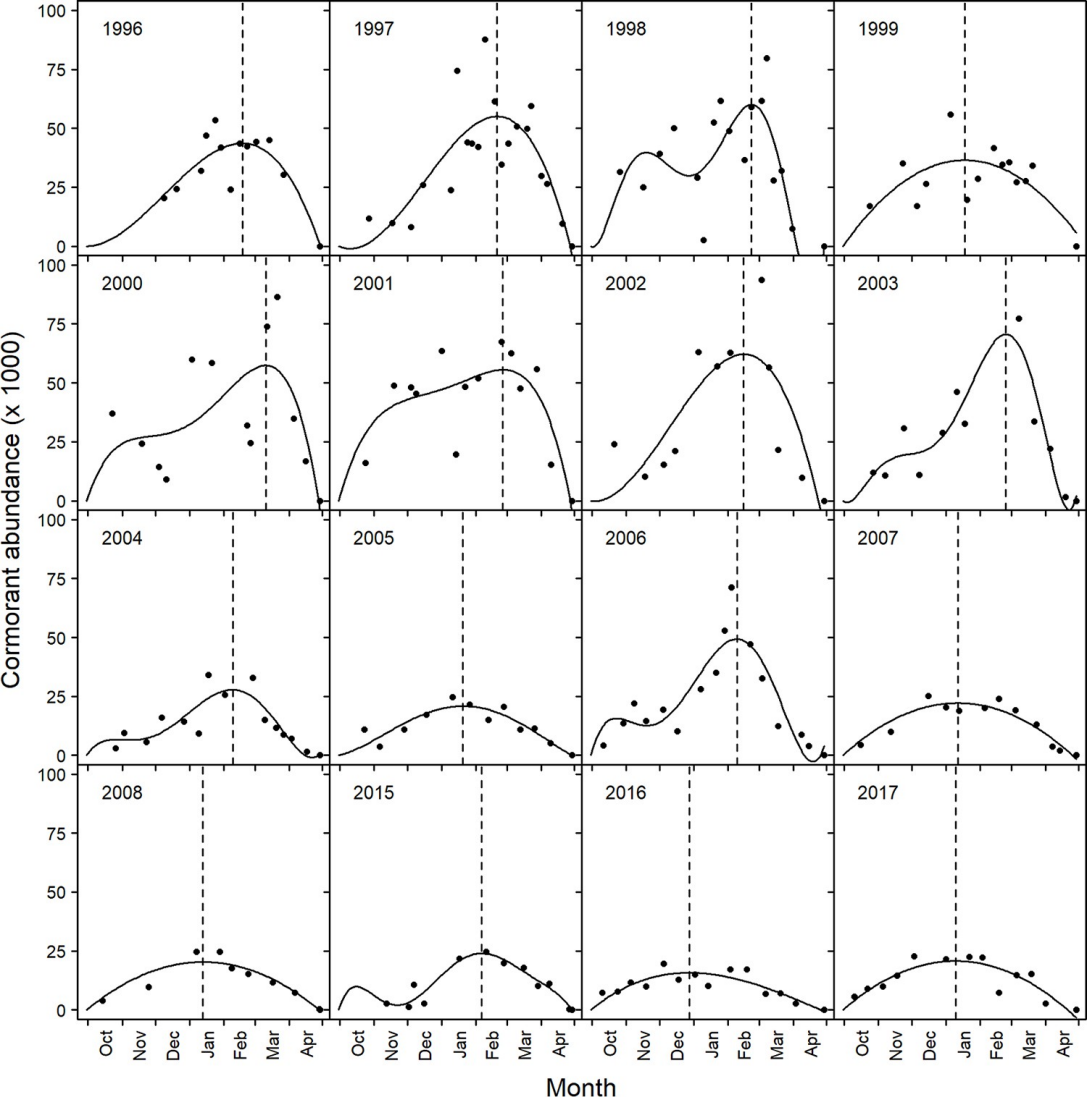
Modeled population trends (solid black line) of survey counts (black points) of cormorants at roosting sites in the Mississippi Delta over the winter seasons (October–April) of 1996–2008 and 2015–2017. Cormorant count was assumed to be zero at the beginning (October 01) and end (April 30) of the winter. Dashed lines indicate the date in which maximum modeled abundance is observed for each year.

**Fig 9:**
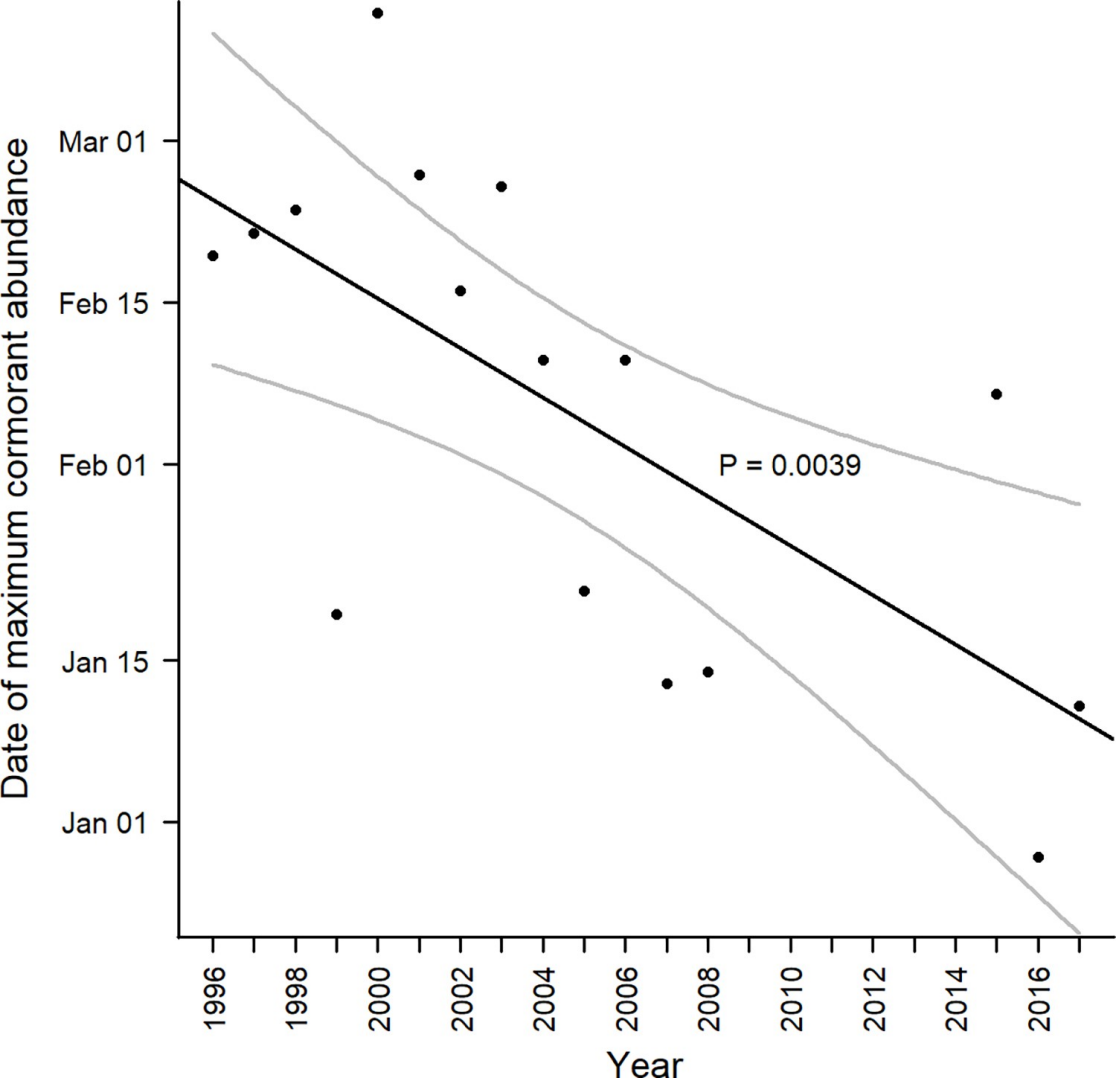
Trend in maximum cormorant abundance in the Mississippi Delta from 1996 through 2017. Dates were estimated from polynomial equations constructed for each year of available data (Fig 8). The black line (+ 95% CI gray lines) represents the significant linear relationship between variables, with a -2.14 slope.

A total of 16 years of aerial survey data were included in abundance modeling of cormorants within the Mississippi Delta (1996–2008, 2015–2017). Although aerial surveys were done during the years of 2009 and 2010, they could not be used in this analysis due to the low sample size of only four and two surveys, respectively. Yearly cormorant abundance trends in the Delta showed either bimodal or unimodal patterns (Fig 8). The latest date associated with maximum cormorant abundance was March 12^th^ in 2000, and the earliest was December 29^th^ in 2016 (Figs 8 and 9). Date of maximum cormorant abundance in the Delta modeled over the last 22 years showed maximum abundance to be occurring progressively earlier (*p* = 0.0039). Specifically, model outputs showed maximum arrival to be occurring 2.14 (95% CI: 0.81–3.47) days earlier every year (Fig 9).

## Discussion

We found that changes in agricultural practices were a significant driver of distribution and abundance of a predatory avian species. Specifically, aquaculture area, and changes in production area, were significant drivers of cormorant distribution and abundance within and between years. Seasonal use and roost-specific use by cormorants was consistent across years, but overall abundance in the region declined with declining production area. This decline in cormorant abundance in the Delta occurred despite a stable or increasing cormorant breeding population over the same time period. We also found the phenology of maximum cormorant abundance in the Delta changed over time, occurring earlier at a critical pre-migratory time period when cormorants are building fat reserves for migration to their northern breeding grounds.

We examined numerous aspects of cormorant ecology in the Delta region using cormorant abundance data collected by the WS-NWRC at roosting sites over almost two decades. Because cormorants are highly reliant on roosting sites [41], and all sites are known and fixed in time and space, these data provide a general census of cormorants at varying time points over the winter season of many years. Such data are not typical while surveying species over such a large spatial scale. Using these data, we were able to address questions pertaining to cormorant distribution and abundance within winter seasons, between years, and in relation to foraging habitat and roost characteristics.

We elected to model cormorant presence and abundance separately. This was done partly to help handle our large data set with many zeros, but also to investigate each process separately. For instance, mechanisms driving where cormorants will roost may not necessarily have the same relationship or significance for the abundance of cormorants at occupied roosts. Seasonal roost use probability and average abundance at roosting sites showed similar patterns, increasing from October through January, and decreasing thereafter (Figs 2 and 4). This relationship follows what has been previously shown with respect to cormorant migration patterns [37]. This trend was in fact what we observed in our population trends for each year (Fig 8) suggesting this has been the long-term pattern of cormorant abundance in this region.

We found the main effect of aquaculture area to have a significant influence on cormorant presence at a roost. As aquaculture area increased within a 23.4 km buffer around a roost, the probability of that roost being used also increases. Most daily activities of cormorants include roosting and foraging [41], therefore it was unsurprising roosts with greater amounts of forage potential have increased probability of use. There was also a significant interaction of aquaculture area and date on cormorant presence at roosting sites. Early in the winter season the area of aquaculture around a roost has less influence on whether cormorants decide to use it (Fig 3). The main effect of aquaculture area on average roost abundance was not a significant predictor. This can be explained by a cross over event linked to the date, which was observed in our interaction term of date and aquaculture area. Early in the winter season aquaculture area has a negative influence on average roost count, however later in the winter aquaculture area has a positive influence (Fig 5). Therefore, the probability of a roost being used was overall positive given the area of aquaculture around the roost whereas the average abundance at the roost was more seasonally dependent. For example, in mid-November the probability of cormorant being present at a given roost was relatively constant regardless of the area of surrounding aquaculture, but the average abundance was likely to be greater at a roost with less aquaculture. However, in mid-March the probability of use and the average abundance were both greater at roosts with more surrounding aquaculture.

The increasing seasonal influence of aquaculture on cormorant roost use and abundance suggests a shift toward aquaculture later in the winter prior to spring migration north [10]. This shift has been observed in food habit studies where the proportion of catfish in cormorant diets increases later in winter [52]. Bioenergetics models of cormorants estimated the number of consumed catfish to be the highest toward the end of the winter season [53]. Another study surveyed cormorants on natural waterbodies and aquaculture ponds in the Delta and found cormorants to use aquaculture proportionally more later in the winter [67]. A possible explanation for this diet shift is that cormorants are taking advantage of the high densities of fish stocked in aquaculture ponds to meet the energy requirements of northly migration [7]. It has also been reported that cormorants found in areas of high aquaculture production had higher omental fat compared to cormorants found away from aquaculture production, which can indicate improved body condition and overall fitness [32].

Given the perceived benefits to cormorants foraging on aquaculture ponds, it is logical to ask why they do not constantly occupy roosts near aquaculture facilities. This is likely due to human intervention through roost harassment and management activities at aquaculture facilities themselves. A primary management strategy aimed at reducing cormorant abundance around aquaculture facilities is roost harassment [68, 69]. This strategy typically involves using pyrotechnics, or other frightening devices and tactics, to push cormorants out of roosts near areas of aquaculture. In general, the goal is to shift cormorant distribution westerly toward the Mississippi river where there has consistently been less aquaculture compared to the east-central Delta [70]. In addition, catfish producers routinely use harassment techniques against cormorants at their farms, including lethal take through the aquaculture depredation order (AQDO; 50 CFR 21.47) [27, 71]. The risk associated with roost harassment, and the risk at the facilities themselves may keep cormorants restricted to using roosts away from aquaculture facilities. However, later in the winter season a tradeoff may occur where the benefit of foraging on aquaculture ponds outweighs the risks.

We predicted the number of nearby roosts would influence cormorant presence, but our results suggest no relationship (Fig 2). This may be confounded by the fact roosts with many neighboring roosts tended to be in the east-central Delta, which also contains a greater area of aquaculture. As we observed however, a shift of use and abundance more toward roosts with greater aquaculture area occurred as the winter season progressed (Figs 3 and 5). This shift based on date may be overwhelming potential effects of neighboring roosts. We did not include the number of neighboring roosts in our count model, as we did not hypothesize a reason for it to influence abundance. We did include roost area as a variable in abundance as larger roosts will have a greater potential capacity to hold cormorants, and this was what we observed in our models (Fig 4). A census of cormorants in the Delta during winters 1991–92 and 1992–93 found primary roosting sites to be larger bodies of water with large stands of roosting trees [43]. We also included year as a variable when modeling average roost abundance because cormorant abundance within the Delta has been dynamic over the last few decades (Fig 8). Average roost abundance has decreased over time, similar to total cormorant abundance in the Delta. However, mean roost abundance seems to have leveled off in the last few years (Fig 4). This pattern was similar to aquaculture acreage within the Delta. Specifically, the loss of aquaculture area in the Delta has slowed, and even shows a slight comeback since 2013 (Fig 6).

Midwest breeding bird survey data showed cormorant abundance has been steadily increasing over the past 30 years, whereas cormorant abundance in the Delta peaked in the early 2000’s and has generally declined thereafter. This observation suggests aquaculture area in the Delta is a driving factor in the abundance of cormorants wintering in the region and is supported by our analysis. Aquaculture area in the Delta accounted for over four times the estimated R^2^ of the model compared to the breeding bird survey index. This is more evident when observing the raw data points with the modeled relationship of breeding bird survey index (Fig 7). Although statistically significant, the breeding bird survey index shows less of a general pattern compared to aquaculture area, but a positive influence of breeding bird survey index is intuitive. As the Midwest breeding population changes, it is expected to cause similar changes in the wintering region of cormorants. Earlier accounts of cormorants in Mississippi suggest cormorants spent little time wintering inland, but rather traveled closer to the coast [31]. However, as aquaculture facilities became a dominant item on Mississippi’s landscape, cormorants may have begun to winter closer to these facilities. Now that aquaculture area has reduced by more than 70% [58], and therefore forage potential has also been reduced, cormorants may be distributing themselves to other regions farther south. We used maximum recorded abundance within a given year, however it is still possible the number of cormorants moving through the Mississippi Flyway is increasing along with the Midwest breeding population, although fewer remain in the Delta region.

Studies investigating changes in migration phenology typically incorporate some measure of arrival or departure dates, such as first observed species occurrence, or some central measure of capture dates [72–74]. How to select a response like this is subject to the species studied and survey methodology used. We used population estimates created from polynomial regression models using abundance data collected over multiple surveys per year. Biologically, this period of maximum abundance corresponds with peak numbers of cormorants using an important stopover location on migration to their breeding grounds. Observation of the model fit, along with the associated R^2^ values indicate this as a reasonable approach for such data (Fig 8). Maximum modeled abundance of cormorants in the Delta during the late 1990s and early 2000s averaged mid-February. However, maximum modeled abundance is currently occurring earlier, approximately in mid-January. Our estimated slope indicated maximum modeled abundance of cormorants to be happening 2.14 days earlier per year, which is similar to other migratory species reported, although significant variation does exist [74, 75]. Given that the rapid decline in abundance in early April appears consistent across years, this change in stopover phenology suggests that cormorants are arriving earlier, staying in the stopover areas longer, but departing northward at a similar time.

Midwinter roost counts are done to gauge the overall abundance of cormorants wintering in the Delta and are typically conducted in early February. Given our results, a midwinter count in mid-January would result in the survey being done approximately when the maximum number of cormorants are inhabiting the Delta. Also, cormorant management could be focused at this time if resources are limited. However, taking action at roosting sites in January is logistically challenging due to the waterfowl hunting season, as many roosts are privately owned by hunting clubs and do not allow access during the hunting season.

**Fig 10:**
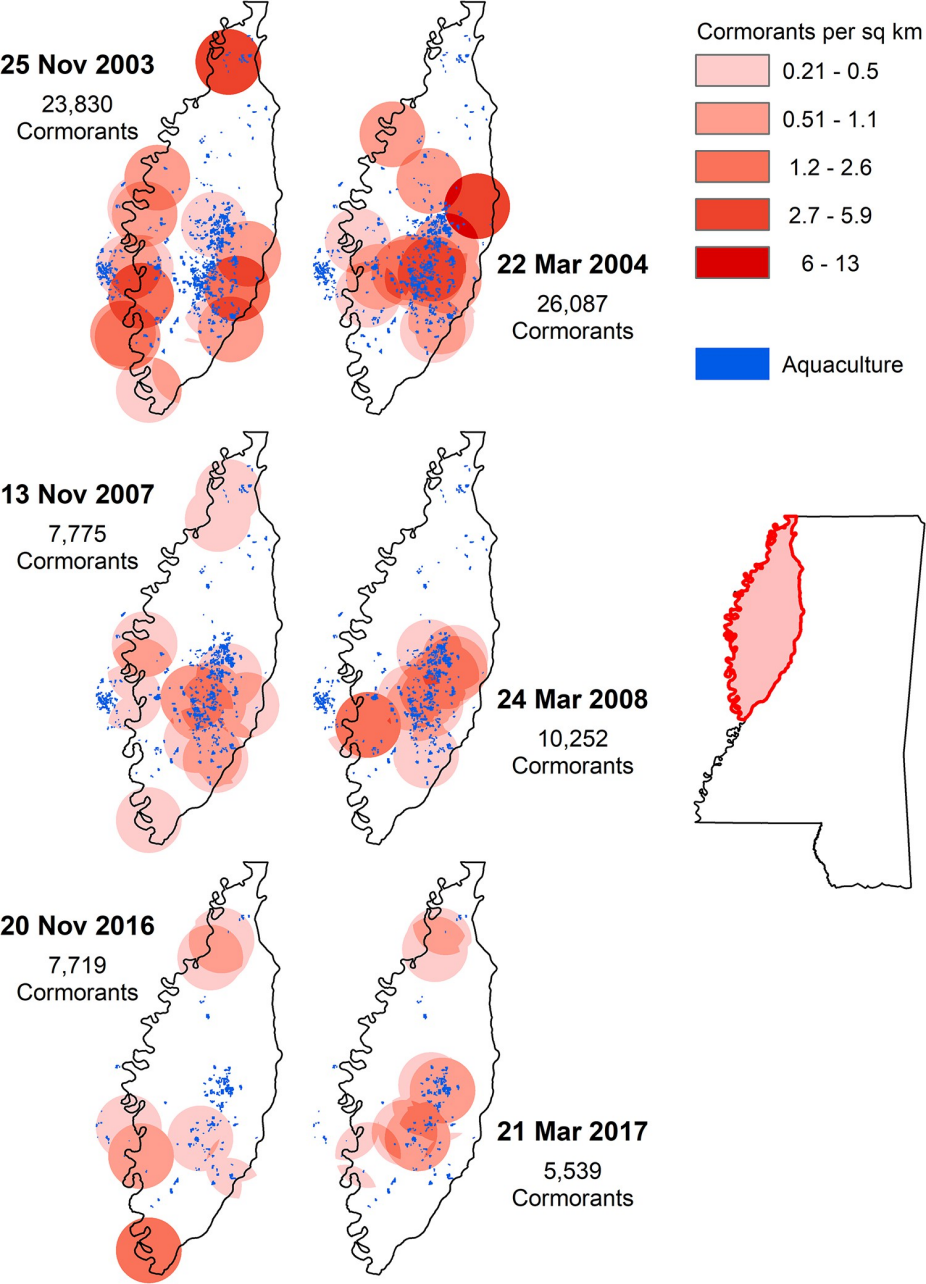
Cormorant density and temporal and spatial distribution in the Mississippi Delta based on aerial surveys of all roosting locations done in November and March during each of the winter seasons of 2003–2004, 2007–2008, and 2016–2017. Densities are based on the number of cormorants at each roost divided by the area within a 23.4 km buffer around each roost. Mississippi state outline and Mississippi Delta outline shapefiles were downloaded from Mississippi Automated Resource Information System (MARIS) at Maris.mississippi.edu. Mississippi state and Mississippi Delta shapefile credit: U.S. Census and MARIS with no use limitations. Aquaculture shapefile was created by manually digitizing aquaculture using Landsat imagery, courtesy of the U.S. Geological Survey. Cormorant densities shapefiles were created using survey data collected by the U.S. Department of Agriculture, Wildlife Services. This figure was created in ArcGIS.

Key findings of this study are shown in Fig 10 which provides a spatial and temporal depiction of cormorant distribution and abundance throughout the Delta, and with respect to aquaculture area. We selected two aerial roost surveys from each of the winter seasons of 2003/04, 2007/08, and 2016/17. For each winter season, we selected one survey from November that represents an early winter period, and one survey from March that represents a late winter period. For each of the six selected surveys we calculated cormorant densities based on each roosts count divided by area of the 23.4 km buffer around each roost. The resulting maps illustrate multiple conclusions worth highlighting. First, the spatial extent in which aquaculture surface area has decreased since the early 2000s is epitomized when examining the maps between years. Second, there is a decreasing annual cormorant abundance in the Delta as seen by the changing density intensities and total cormorants at similar times among winters. The maximum aerial survey count was 60,108 in the winter of 2003/04, 19,539 in the winter 2007/08, and 15,271 in 2016/17. Third, cormorants show a seasonal shift in distribution in relation to aquaculture, with a tendency to become less concentrated on aquaculture areas during the early winter survey period compared to the late winter survey period.

## Conclusion

Our research indicates that large-scale changes in an agricultural commodity, in this case aquaculture, can drive wintering abundance and distribution of a migrating bird species. Use of aquaculture was not constant but rather seasonally dependent, suggesting shifts in resource selection occur based on fluctuating energy demands such as those related to migration. Furthermore, peak wintering abundance of cormorants has been shifting earlier over the past two decades suggesting large scale environmental influences such as climate change may be influencing wintering phenology in this species. These factors may interact to cause profound changes in not only impacts to aquaculture by cormorants, but biology and behavior of the birds themselves.

## Supporting information

S1 Dataset Data used in the analyses of cormorant presence and abundance at roosting sites in the Mississippi Delta. (XLSX)

S2 Dataset Yearly data of maximum cormorant abundance and the total area of aquaculture present within the Mississippi Delta. (XLSX)

S3 Dataset Aerial survey data of total cormorants counted at all surveyed roosts within the Mississippi Delta. (XLSX)
